# Linear-time algorithms for phylogenetic tree completion under Robinson–Foulds distance

**DOI:** 10.1186/s13015-020-00166-1

**Published:** 2020-04-13

**Authors:** Mukul S. Bansal

**Affiliations:** 1 Department of Computer Science and Engineering, University of Connecticut, 371 Fairfield Way, Storrs, USA; 2 Institute for Systems Genomics, University of Connecticut, Storrs, USA

**Keywords:** Phylogenetics, Distance measures, Robinson–Foulds distance, Optimal phylogenetic tree completion

## Abstract

**Background:**

We consider two fundamental computational problems that arise when comparing phylogenetic trees, rooted or unrooted, with non-identical leaf sets. The first problem arises when comparing two trees where the leaf set of one tree is a proper subset of the other. The second problem arises when the two trees to be compared have only partially overlapping leaf sets. The traditional approach to handling these problems is to first restrict the two trees to their common leaf set. An alternative approach that has shown promise is to first *complete* the trees by adding missing leaves, so that the resulting trees have identical leaf sets. This requires the computation of an optimal completion that minimizes the distance between the two resulting trees over all possible completions.

**Results:**

We provide optimal linear-time algorithms for both completion problems under the widely-used Robinson–Foulds (RF) distance measure. Our algorithm for the first problem improves the time complexity of the current fastest algorithm from quadratic (in the size of the two trees) to linear. No algorithms have yet been proposed for the more general second problem where both trees have missing leaves. We advance the study of this general problem by proposing a useful restricted version of the general problem and providing optimal linear-time algorithms for the restricted version. Our experimental results on biological data sets suggest that completion-based RF distances can be very different compared to traditional RF distances.

## Background

A *phylogenetic tree*, or *phylogeny*, is a uniquely leaf-labeled tree that shows the evolutionary relationships between different biological entities, generally either species or genes. Phylogenies may be either rooted or unrooted. The leaf nodes of a phylogeny represent the extant set of entities on which the phylogeny is built, while internal nodes represent hypothetical ancestors. The comparison of different phylogenetic trees is one of the most fundamental tasks in evolutionary biology and computational phylogenetics. Many biologically relevant distance or similarity measures have been defined in the literature for the case when the two phylogenies to be compared have the same leaf set. These include the widely used Robinson–Foulds distance [1], triplet and quartet distances [2, 3], nearest neighbor interchange (NNI) and subtree prune and regraft (SPR) distances [4–6], maximum agreement subtrees [7–9], nodal distance [10], geodesic distance [11] and several others. Often, however, this comparison involves two trees that have non-identical leaf sets. The need to compare trees that do not have identical leaf sets arises naturally in several situations: For instance, algorithms for computing phylogenetic supertrees are typically based on comparing input trees on partial leaf sets with candidate supertrees on the complete leaf set [12–16]. Likewise, searching for phylogenies similar to a query tree in a phylogenetic database [17–20] and clustering of phylogenetic trees [21] often involve comparisons between trees with only partially overlapping leaf sets.

**Fig. 1:**
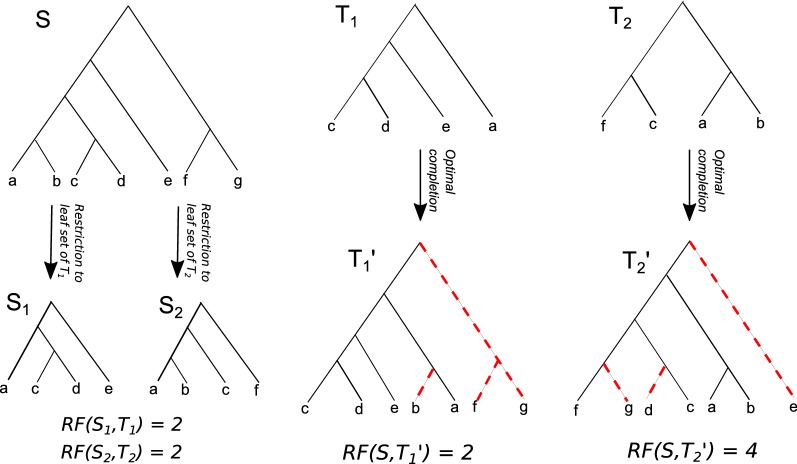
RF(−) and RF(+) distances. This figure illustrates the difference between the traditional (RF(−)) and RF(+) distance measures when applied to trees with partially overlapping leaf sets. In this example, the leaf sets of $T_1$ and $T_2$ are a subset of the leaf set of *S*. To compute the RF(−) distance between $T_1$ and *S*, we must first restrict *S* to the leaf set of $T_1$, resulting in tree $S_1$. The RF(−) distance between *S* and $T_1$ is thus $RF (S_1, T_1)$, which is 2. Likewise, to compute the RF(−) distance between $T_2$ and *S*, we must first restrict *S* to the leaf set of $T_2$, resulting in tree $S_2$. The RF(−) distance between *S* and $T_2$ is thus $RF (S_2, T_2)$, which is also 2. In contrast, to compute the RF(+) distance between $T_1$ and *S*, we must first compute an optimal completion of $T_1$ on the leaf set of *S* (denoted by the dashed red lines), resulting in tree $T_1'$. The RF(+) distance between *S* and $T_1$ is thus $RF (S, T_1')$, which is 2. Likewise, to compute the RF(+) distance between $T_2$ and *S*, we must first compute an optimal completion of $T_2$ on the leaf set of *S*, resulting in tree $T_2'$. The RF(+) distance between *S* and $T_2$ is thus $RF (S, T_2')$, which is 4. Observe that while both $T_1$ and $T_2$ are equidistant from *S* under RF(−), computing the RF(+) distances reveals that $T_1$ is more similar to *S* than is $T_2$

The traditional approach to comparing two phylogenies on non-identical leaf sets is to first restrict the two phylogenies to their common leaf set and then apply one of the distance or similarity measures that compare two trees on the same leaf set. However, an alternative, and perhaps more useful, approach to comparing trees with non-identical taxa is to *fill-in* or *complete* the two trees to be compared with the leaves missing from each, resulting in two trees on the same leaf set, and then apply the distance or similarity measure. This completion based approach is especially desirable when used with the Robinson–Foulds (RF) distance measure [1], the most commonly used distance measure in evolutionary biology. Indeed, several important biological applications would directly benefit from the use of this completion-based RF distance, such as the construction of majority-rule(+) supertrees [22–25], construction of Robinson–Foulds supertrees [13, 14, 26], phylogenetic database search [17–20], and clustering of phylogenetic trees [21]. To distinguish between the two methods for computing RF distance between two trees with non-identical leaf sets, we refer to the completion-based RF distance as RF(+) distance and to the traditional pruning-based RF distance as RF(−). Figure 1 shows an example of two trees with partially overlapping leaf sets and these two ways of computing the RF distance between them.

### Previous work

The idea of a completion-based RF(+) distance was proposed at least a decade ago. Cotton and Wilkinson were among the first to propose such a distance measure in their seminal paper describing majority-rule supertrees [22]. Specifically, they defined two types of majority-rule supertrees: majority-rule(−) and majority-rule(+) supertrees. The majority-rule(−) supertrees were based on traditional RF(−) distances between trees, while majority-rule(+) supertrees were based on completion-based RF(+) distances. Majority-rule(+) supertrees and its variants have been shown to have many desirable properties [27] and there have been efforts to develop exact (ILP-based) and heuristic methods for computing majority-rule(+) supertrees [23, 25]. Though these methods only work for small datasets, they have been shown to result in biologically meaningful supertrees [23]. The paper by Kupczok [25] characterizes the RF(+) distance in the case when the leaf set of one tree is a subset of the leaf set of the other in terms of incompatible splits between the two trees, but does not provide an efficient algorithm for computing this distance or for computing an actual completion. More recently, Christensen et al. [28] provided an $O(n^2)$ time algorithm for the case when the leaf set of one tree is a subset of the leaf set of the other and applied the algorithm to compute optimal completions for gene trees with respect to a species tree. To the best of our knowledge, no algorithms (polynomial time or otherwise) currently exist for the general problem where the two trees have only partially overlapping leaf sets, or for any of its variants.

### Our contribution

In this work, we address an important gap in the algorithmics of phylogenetic tree comparison. Specifically, we provide the first optimal, linear-time algorithms for two fundamental computational problems that arise when comparing phylogenetic trees with non-identical leaf sets. For the first problem, which arises when computing the RF(+) distance between two binary trees where the leaf set of one tree is a proper subset of the other, we improve upon the time complexity of the previous fastest algorithm for this problem by a factor of *n*, where *n* is the number of leaves in the larger of the two trees. For the second problem, which is a generalization of the first and arises when computing the RF(+) distance between two binary trees that have only partially overlapping leaf sets, we show that the default problem formulation can result in tree completions that are unsupported by the original input trees, propose a modification of the problem formulation that corrects this deficiency, and provide optimal linear-time algorithms for the modified problem. Crucially, no polynomial time algorithms currently exist for the default formulation of the second problem, and our modified problem formulation can be viewed as a useful restricted version of the general problem. Our algorithms are easy to understand and implement, work for both rooted and unrooted trees, and are scalable to the entire tree of life. These algorithms can be applied wherever phylogenetic distances must be computed between trees with non-identical leaf sets and enable new kinds of phylogenetic and comparative analyses that have been computationally infeasible.

We implemented our algorithm for the first problem and applied it to three published biological supertree data sets to study how RF(+) distances differ from RF(−) distances in practice. For each data set, we ordered the input trees according to their RF(+) and RF(−) distances to a precomputed supertree and measured how often the relative pairwise ranking between any pair of input trees differs between the two rankings. We found a large number of such pairs for each data set, demonstrating, for the first time, that using the RF(+) distance can result in very different relative estimates of phylogenetic distances compared to using the RF(−) distance.

RF(+) distances have several desirable properties compared to RF(−) distances. For instance, the set of possible values RF(+) distance can take ranges from 0 to about twice the size of the *union* of the leaf sets of the two trees, while for RF(−) distance this range is only from 0 to about twice the size of the *intersection* of the two leaf sets. Thus, RF(+) distances have significantly more discriminatory power than RF(−) distances. In applications such as median supertree construction, RF(+) distance has the distinct advantage that each input tree gets an equal “vote” in the supertree construction since all input trees contribute an RF distance within the same range. With RF(−) distances, larger trees can contribute much more to the total distance than smaller trees. Finally, in computing RF(−) distances we ignore the additional topological information provided by leaves that are present in only one tree, while RF(+) distance makes complete use of the information in the topologies of the two trees. RF(+) distances thus make more efficient use of the available information. Despite these advantages, RF(+) distances have not been applied in practice due to unavailability of efficient algorithms. In contrast, RF(−) distances can be computed in time linear in the sizes (number of leaves) of the input trees. Our new algorithms address this discrepancy by making it equally computationally efficient to compute RF(+) distances.

## Preliminaries and problem definitions

Given a tree *T*, we denote its node set, edge set, and leaf set by *V*(*T*), *E*(*T*), and ${{\,\mathrm{\textit{Le}}\,}}(T)$, respectively. The set of all non-leaf (i.e., internal) nodes of *T* is denoted by *I*(*T*).

If *T* is rooted, the root node of *T* is denoted by ${{\,\mathrm{\textit{rt}}\,}}(T)$, the parent of a node $v \in V(T)$ by ${{\,\mathrm{\textit{pa}}\,}}_T(v)$, its set of children by ${{\,\mathrm{\textit{Ch}}\,}}_T(v)$, and the (maximal) subtree of *T* rooted at *v* by *T*(*v*). If two nodes in *T* have the same parent, they are called *siblings* of each other. The *least common ancestor*, denoted $\textit{lca}_{T}(L)$, of a set $L \subseteq {{\,\mathrm{\textit{Le}}\,}}(T)$ in *T* is defined to be the node $v \in V(T)$ such that $L \subseteq {{\,\mathrm{\textit{Le}}\,}}(T(v))$ and $L \not \subseteq {{\,\mathrm{\textit{Le}}\,}}(T(u))$ for any child *u* of *v*. A rooted tree is *binary* if all of its internal nodes have exactly two children, while an unrooted tree is *binary* if all its nodes have degree either 1 or 3. Throughout this work, the term *tree* refers to binary trees with uniquely labeled leaves.

Let *T* be a rooted or unrooted tree. Given a set $L \subseteq {{\,\mathrm{\textit{Le}}\,}}(T)$, let ${\overline{T}}$ be the subtree of *T* with leaf set *L*. We define the *leaf induced subtreeT*[*L*] of *T* on leaf set *L* to be the tree obtained from ${\overline{T}}$ by successively removing each non-root node of degree two and adjoining its two neighbors.

### **Definition 1**

(Completion of a tree) Given a tree *T* and a set $L'$ such that ${{\,\mathrm{\textit{Le}}\,}}(T) \subseteq L'$, a *completion* of *T* on $L'$ is a tree $T'$ such that ${{\,\mathrm{\textit{Le}}\,}}(T') = L'$ and $T'[{{\,\mathrm{\textit{Le}}\,}}(T)] = T$.

If *T* is a rooted tree, for each node $v \in V(T)$, the *clade*$C_T(v)$ is defined to be the set of all leaf nodes in *T*(*v*); i.e. $C_T(v) = {{\,\mathrm{\textit{Le}}\,}}(T(v))$. We denote the set of all clades of a rooted tree *T* by ${{\,\mathrm{\textit{Clade}}\,}}(T)$. This concept can be extended to unrooted trees as follows. If *T* is an unrooted tree, each edge $(u,v) \in E(T)$ defines a partition of the leaf set of *T* into two disjoint subsets ${{\,\mathrm{\textit{Le}}\,}}(T_u)$ and ${{\,\mathrm{\textit{Le}}\,}}(T_v)$, where $T_u$ is the subtree containing node *u* and $T_v$ is the subtree containing node *v*, obtained when edge (*u*, *v*) is removed from *T*. The partition induced by any edge $(u,v) \in E(T)$ is called a *split* and is represented by the set $\{{{\,\mathrm{\textit{Le}}\,}}(T_u), {{\,\mathrm{\textit{Le}}\,}}(T_v)\}$. The set of all splits in an unrooted tree *T* is denoted by ${{\,\mathrm{\textit{Split}}\,}}(T)$.

The *symmetric difference* of two sets *A* and *B*, denoted by $A \Delta B$, is the set $(A \setminus B) \cup (B \setminus A)$.

### **Definition 2**

(Robinson–Foulds distance) The *Robinson–Foulds (RF) distance*, $RF (S, T)$, between two trees *S* and *T* is defined to be $|{{\,\mathrm{\textit{Clade}}\,}}(S) \Delta {{\,\mathrm{\textit{Clade}}\,}}(T)|$ if *S* and *T* are rooted trees, and $|{{\,\mathrm{\textit{Split}}\,}}(S) \Delta {{\,\mathrm{\textit{Split}}\,}}(T)|$ if *S* and *T* are unrooted trees.

Let *S* and *T* be two trees. Without loss of generality, we will assume that $|{{\,\mathrm{\textit{Le}}\,}}(T)| \le |{{\,\mathrm{\textit{Le}}\,}}(S)|$. When ${{\,\mathrm{\textit{Le}}\,}}(S) \ne {{\,\mathrm{\textit{Le}}\,}}(T)$, there are two possible scenarios: (1) ${{\,\mathrm{\textit{Le}}\,}}(T) \subsetneq {{\,\mathrm{\textit{Le}}\,}}(S)$, i.e., the leaf set of *T* is a proper subset of the leaf set of *S*, and (2) ${{\,\mathrm{\textit{Le}}\,}}(S) \cap {{\,\mathrm{\textit{Le}}\,}}(T) \subsetneq {{\,\mathrm{\textit{Le}}\,}}(T)$, i.e., each of *S* and *T* contains leaves not found in the other. Based on these two scenarios, and depending on whether the two trees are rooted or unrooted, we define the following four problems.

### **Problem 1**

(Rooted One-Tree RF(+) (ROT-RF(+))) *Given two rooted treesSandTsuch that*${{\,\mathrm{\textit{Le}}\,}}(T) \subseteq {{\,\mathrm{\textit{Le}}\,}}(S)$, *compute a completion*$T'$*ofTon*${{\,\mathrm{\textit{Le}}\,}}(S)$*such that*$RF (S, T')$ is minimized.

### **Problem 2**

(Unrooted One-Tree RF(+) (UOT-RF(+))) *Given two unrooted treesSandTsuch that*${{\,\mathrm{\textit{Le}}\,}}(T) \subseteq {{\,\mathrm{\textit{Le}}\,}}(S)$, *compute a completion*$T'$*ofTon*${{\,\mathrm{\textit{Le}}\,}}(S)$*such that*$RF (S, T')$*is minimized.*

### **Problem 3**

(Rooted RF(+) (R-RF(+))) *Given two rooted treesSandT*, *compute a completion*$S'$*ofSon*${{\,\mathrm{\textit{Le}}\,}}(S) \cup {{\,\mathrm{\textit{Le}}\,}}(T)$*and a completion*$T'$*ofTon*${{\,\mathrm{\textit{Le}}\,}}(S) \cup {{\,\mathrm{\textit{Le}}\,}}(T)$*such that*$RF (S', T')$*is minimized.*

### **Problem 4**

(Unrooted RF(+) (U-RF(+))) *Given two unrooted treesSandT*, *compute a completion*$S'$*ofSon*${{\,\mathrm{\textit{Le}}\,}}(S) \cup {{\,\mathrm{\textit{Le}}\,}}(T)$*and a completion*$T'$*ofTon*${{\,\mathrm{\textit{Le}}\,}}(S) \cup {{\,\mathrm{\textit{Le}}\,}}(T)$*such that*$RF (S', T')$*is minimized.*

We show how to solve Problems 1 and 2 in *O*(|*V*(*S*)|) time. As we will see later, Problems 3 and 4 can actually lead to unsupported completions. We will therefore define meaningful variants of Problems 3 and 4 (requiring only a slight variation on the original problems) and show how to solve them in $O(|V(S)| + |V(T)|)$ time. For the purposes of complexity analysis, we will assume that the leaves of *S* and *T* are labeled by integers from the set $\{1, \ldots , |{{\,\mathrm{\textit{Le}}\,}}(S) \cup {{\,\mathrm{\textit{Le}}\,}}(T)|\}$. However, our algorithms work even if the leaf labels are arbitrary, and universal hashing [29] or perfect hashing [30] can be used to guarantee expected $O(|V(S)| + |V(T)|)$ time complexity.

## A linear-time algorithm for ROT-RF(+)

To solve the ROT-RF(+) problem, our algorithm starts with the trees *S* and *T* and modifies *T* by adding to it, according to a particular scheme, the leaves from ${{\,\mathrm{\textit{Le}}\,}}(S) \setminus {{\,\mathrm{\textit{Le}}\,}}(T)$. The completed tree thus produced, denoted by $T'$, will be such that $RF (S, T')$ is minimized.

We define $\textit{Tree-Add}(T, v, X)$ to be the tree obtained from *T* by attaching to it a tree *X*, where ${{\,\mathrm{\textit{Le}}\,}}(X) \cap {{\,\mathrm{\textit{Le}}\,}}(T) = \emptyset$, as follows: If *v* is not the root of *T*, then attach *X* onto the edge $({{\,\mathrm{\textit{pa}}\,}}(v), v)$ (by subdividing $({{\,\mathrm{\textit{pa}}\,}}(v), v)$ into two edges) such that ${{\,\mathrm{\textit{rt}}\,}}(X)$ becomes the sibling of the node $v \in V(T)$. If *v* is the root of *T*, then $\textit{Tree-Add}(T, v, X)$ is the tree obtained by creating a new root node and setting *v* and ${{\,\mathrm{\textit{rt}}\,}}(X)$ as its two children.

The main idea behind our algorithm can be illustrated by the following simple example. Suppose the given trees *S* and *T* are such that ${{\,\mathrm{\textit{Le}}\,}}(S) = {{\,\mathrm{\textit{Le}}\,}}(T) \cup \{l\}$. The goal is to add this leaf *l* to *T* so as to minimize the RF distance. Let *v* denote the sibling of *l* in *S*. Let *u* denote the node $\textit{lca}_{T}({{\,\mathrm{\textit{Le}}\,}}(S(v)))$. As we will prove later, $T' = \textit{Tree-Add}(T, u, l)$ must be an optimal completion for *T*. Our algorithm extends this idea to the case when *T* has multiple missing leaves. A description of the algorithm follows: 
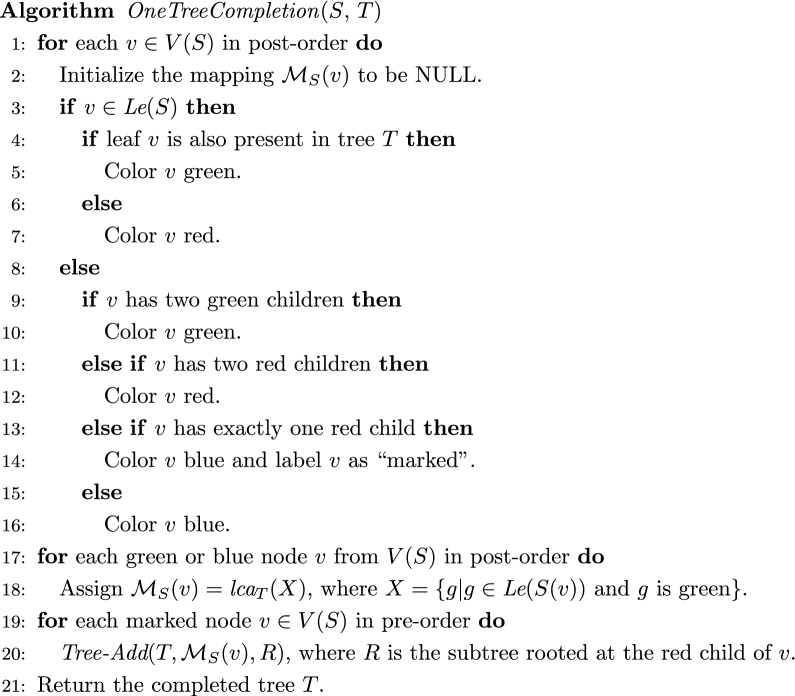

**Fig. 2:**
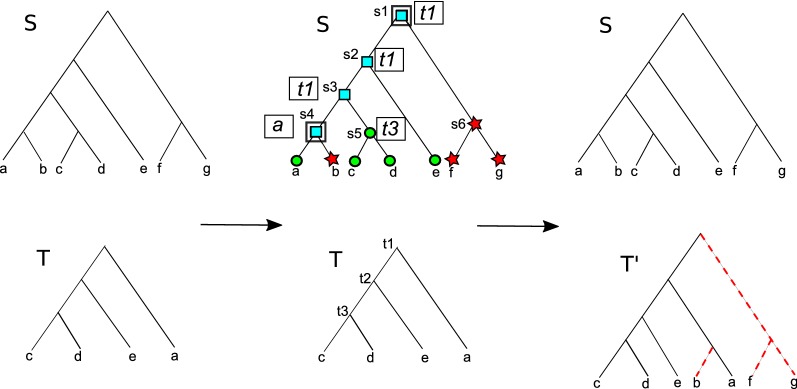
Algorithm for ROT-RF(+). Given *S* and *T* as shown in the left column of the figure, Algorithm *OneTreeCompletion* first colors each node of *S* either green (circles), red (stars), or blue (squares) as shown in the middle column of the figure. A node is colored green if all leaves in the subtree rooted at that node are present in both *S* and *T*, red if all leaves in that subtree are present only in *S*, and blue if that subtree has both green and red descendants. If a blue node *v* has exactly one red child, then it is “marked”. In this example, $s_1$ and $s_4$ are marked nodes, highlighted in the figure by the double perimeter around the blue (square) nodes. The algorithm then computes the LCA mapping, defined to be $\textit{lca}_{T}({{\,\mathrm{\textit{Le}}\,}}(S(v)) \cap {{\,\mathrm{\textit{Le}}\,}}(T))$, for each green or blue node *v* of *S*. These LCA mappings appear in the square boxes on *S* in the middle column. The algorithm then performs a pre-order traversal of *S*, grafting copies of the red subtrees at each marked node onto the appropriate edges of *T*. The grafted subtrees are shown using dashed red lines on $T'$ in the right column. Tree $T'$ is an optimal completion of *T* on ${{\,\mathrm{\textit{Le}}\,}}(S)$

Figure 2 illustrates the algorithm through an example. Next, we prove the correctness and analyze the time complexity of this algorithm. We need the following additional definitions:

### **Definition 3**

(Matched clade) Given any two rooted trees *A* and *B* on the same leaf set, and $v \in V(A)$, we say that clade $C_A(v)$ has a *match* in *B* if ${{\,\mathrm{\textit{Clade}}\,}}(B)$ contains $C_A(v)$.

### **Definition 4**

(Matchable clade of *S*) Given any $v \in I(S)$, we call the clade $C_S(v)$*matchable* if there exists some completion of *T* on ${{\,\mathrm{\textit{Le}}\,}}(S)$ that contains the clade $C_S(v)$.

The correctness of Algorithm *OneTreeCompletion* follows from the following lemma.

### **Lemma 1**

*Let*$T'$*denote the completion ofTreturned by Algorithm* *OneTreeCompletionon treesSandT*. *Let*$T^{*}$*denote an optimal completion ofTon*${{\,\mathrm{\textit{Le}}\,}}(S)$*that minimizes*$RF (S, T^*)$. *Then,*$RF (S, T') = RF (S, T^*)$, *implying that*$T'$*is a solution for the ROT-RF(+) problem.*

### *Proof*

It suffices to show that $T'$ maximizes the number of matched clades $C_S(v)$, for $v \in V(S)$.

Observe that Algorithm *OneTreeCompletion* partitions *V*(*S*) into three sets according to the color assigned to each node: red, green, or blue. We will consider these three sets of nodes separately.

*Case 1: Red nodes.* All maximal subtrees in *S* that contain only red nodes are included as is in the completed tree $T'$. Thus, if *v* is a red node then $C_S(v)$ has a match in $T'$. Thus, $T'$ maximizes the number of matched clades $C_S(v)$ over all red *v*.

*Case 2: Green nodes.* We claim that if *v* is green and $C_S(v)$ does not have a match in $T'$ then it must be unmatchable. Suppose $C_S(v)$ has a match in *T*, and let $u \in V(T)$ be such that $C_S(v) = C_T(u)$. Observe that the clade $C_T(u)$ must also appear in $T'$ since no blue node $x \in V(S)$ will be such that ${\mathcal {M}} _{S}(x) \in V(T(u))$. This implies that if $C_S(v)$ has a match in *T* then $C_S(v)$ must also have a match in $T'$. In other words, if $C_S(v)$ does not have a match in $T'$ then $C_S(v)$ cannot have a match in *T*. Now, since $C_S(v)$ only contains leaves that are already present in *T*, no completion of *T* on ${{\,\mathrm{\textit{Le}}\,}}(S)$ can create clade $C_S(v)$ if $C_S(v)$ is not already present in ${{\,\mathrm{\textit{Clade}}\,}}(T)$. Thus, if $C_S(v)$ has no match in *T* then $C_S(v)$ must be unmatchable. This proves our claim, and so $T'$ must maximize the number of matched clades $C_S(v)$ for green *v*.

*Case 3: Blue nodes.* We claim that if *v* is blue and $C_S(v)$ does not have a match in $T'$ then it must be unmatchable. Let $C'_S(v)$ denote the set containing only the green nodes from $C_S(v)$. We will say that clade $C_S(v)$ has a partial-match in *T* if and only if $C'_S(v) \in {{\,\mathrm{\textit{Clade}}\,}}(T)$. Suppose $C_S(v)$ has a partial-match in *T*, and let *u* be the node from *T* for which $C_T(u) = C'_S(v)$ (note that, in fact, $u = {\mathcal {M}} _{S}(v)$). Observe that any marked node $x \in V(S(v))$ must be such that ${\mathcal {M}} _S(x) \in V(T(u))$. This implies that Algorithm *OneTreeCompletion* adds all the maximal red subtrees within *S*(*v*) (i.e., subtrees rooted at a red child of a marked node in *S*(*v*)) to one or more of the edges in the set $\{({{\,\mathrm{\textit{pa}}\,}}(t), t) | t \in T(u)\}$. Moreover, since $C_T(u) = C'_S(v)$, none of the other marked nodes $y \in V(S) \setminus V(S(v))$ can be such that ${\mathcal {M}} _S(y) \in V(T(u))$. Thus, there must be a node $u' \in T'$ for which $C_{T'}(u') = C_T(u) \cup \{r | \text {} r \text {is a red leaf from} S(v)\}$, and so $C_S(v)$ must have a match in $T'$. Consequently, if $C_S(v)$ has a partial-match in *T* then $C_S(v)$ must have match in $T'$. In other words, if $C_S(v)$ does not have a match in $T'$ then $C_S(v)$ cannot have a partial-match in *T*.

Now, suppose $v \in V(S)$ is such that $C_S(v)$ has no partial-match in *T*. Since $C'_S(v)$ only contains leaves that are already present in *T*, and there exists no node $u \in V(T)$ for which $C_T(u) = C'_S(v)$, no completion of *T* on ${{\,\mathrm{\textit{Le}}\,}}(S)$ can create clade $C_S(v)$. Thus, if $C_S(v)$ has no partial-match in *T* then $C_S(v)$ must be unmatchable. This proves our claim, and so $T'$ must maximize the number of matched clades $C_S(v)$ for blue *v*.

In summary, the tree $T'$ maximizes the number of matched clades for each of the three sets into which *V*(*S*) is partitioned, thereby maximizing the number of matched clades over all of *V*(*S*). Hence, $T'$ must be a solution for the ROT-RF(+) problem. $\square$

### **Theorem 1**

*Algorithm* *OneTreeCompletionsolves the ROT-RF(+) problem inO*(|*V*(*S*)|) *time.*

### *Proof*

Lemma 1 establishes that Algorithm *OneTreeCompletion* solves the ROT-RF(+) problem. It therefore suffices to show that this algorithm can be implemented in *O*(|*V*(*S*)|) time. We consider the complexity of each of the three ‘for’ loops separately.

The ‘for’ loop of lines 1 through 16 executes a single post-order traversal of the tree *S*, and so lines 2 through 16 are executed a total of *O*(|*V*(*S*)|) times. Each of the lines 2 through 16, except for line 16, clearly requires only *O*(1) time per iteration. Line 16 can also be executed in *O*(1) time after an *O*(|*S*|) preprocessing step to construct a lookup table that enables *O*(1) time lookup of whether a given leaf label from *S* occurs in tree *T* as well. This lookup table can be easily implemented using an array since the leaves of *S* (and *T*) are uniquely labeled by integers from the set $\{1, \ldots , |{{\,\mathrm{\textit{Le}}\,}}(S)|\}$. The indices of the array correspond to the leaf labels, and the entries correspond to whether the corresponding leaf appears only in *S* or in both *T* and *S*. Such an array can be constructed using a single traversal through the leaf sets of *S* and *T*. Even if the leaves have arbitrary labels, *O*(|*S*|) preprocessing time and expected *O*(1) lookup time can be achieved through hashing [29].

Line 18 is executed a total of *O*(|*V*(*S*)|) times through the ‘for’ loop on line 17. After an *O*(|*V*(*T*)|) preprocessing step on *T*, the least common ancestor of any pair of nodes from *V*(*T*) can be computed in constant time [31]. For any internal node *v* considered in the ‘for’ loop on line 17, observe that $\textit{lca}_{T}(X)$, where $X = \{g | g \in {{\,\mathrm{\textit{Le}}\,}}(S(v)) \text { and g is green}\}$ is equivalent to $\textit{lca}_{T}(Y)$, where $Y = \{{\mathcal {M}} _S(g) | g \in {{\,\mathrm{\textit{Ch}}\,}}_S(v) \text { and g is not red}\}$. Thus, computing the least common ancestor mapping for any *v* (in line 18) is equivalent to computing the least common ancestor of the mappings of its (up to two) blue or green children. Thus, after an $O(|{{\,\mathrm{\textit{Le}}\,}}(T)|)$ preprocessing step on *T* to enable fast least common ancestor computation [31], each execution of line 18 requires only *O*(1) time. This gives a total time complexity of *O*(|*V*(*S*)|) for lines 17 and 18.

The ‘for’ loop on line 19 executes line 20 a total of *O*(|*V*(*S*)|) times. For a marked node *v*, line 20 requires *O*(|*V*(*R*)|) time, where *R* is the subtree rooted at the red child of *v*, to copy over the subtree *R* to *T*. Since each such *R* is disjoint from the others, over all possible marked nodes *v*, the total number of nodes in all the corresponding *R*s is bounded by *O*(|*V*(*S*)|). Thus, the total time complexity of lines 19 and 20 is *O*(|*V*(*S*)|).

Finally, line 21 requires *O*(|*V*(*S*)|) time to write the completed version of *T*. The total time complexity is thus *O*(|*V*(*S*)|). $\square$

Note that Algorithm *OneTreeCompletion* computes a single optimal completion, and that optimal completions need not be unique.

## Solving UOT-RF(+) in linear time

An unrooted tree can be converted into a rooted tree by adding a root node on a chosen edge (thereby splitting the chosen edge into two edges, with the two end points of the chosen edge becoming the two children of the root node). Thus, if the unrooted tree has *e* edges then there are *e* ways to root that tree, with each of the *e* ways resulting in a different rooted tree.

If *S* and *T* are unrooted trees then we will show how to compute an optimal completion of *T* on ${{\,\mathrm{\textit{Le}}\,}}(S)$ by using Algorithm *OneTreeCompletion* on appropriately rooted versions of *S* and *T*. The following observation establishes a direct relationship between the RF distance between two unrooted trees on the same leaf set and the RF distance between appropriately rooted versions of the two unrooted trees. This observation is also proved in [14].

### **Observation 1**

*LetPandQbe unrooted trees on the same leaf set, andlbe any leaf node (common toPandQ*). *Let*${\widehat{P}}$*be obtained by rootingPon the edge connectinglto the rest ofP*, *and*${\widehat{Q}}$*be obtained by rootingQon the edge connectinglto the rest ofQ*. *Then,*$RF (P, Q) = RF ({\widehat{P}}, {\widehat{Q}})$.

### *Proof*

Consider any edge $(u,v) \in E(P)$. We will use $P_u$ to denote the subtree containing node *u* and $P_v$ to denote the subtree containing node *v*, obtained when edge (*u*, *v*) is removed from *P*. Edge (*u*, *v*) defines the split $\{{{\,\mathrm{\textit{Le}}\,}}(P_u), {{\,\mathrm{\textit{Le}}\,}}(P_v)\}$ in *P*. We define a bijection $f:{{\,\mathrm{\textit{Split}}\,}}(P) \rightarrow {{\,\mathrm{\textit{Clade}}\,}}({\widehat{P}}) \setminus \{l, {{\,\mathrm{\textit{rt}}\,}}(P)\}$ from splits in *P* to clades in ${\widehat{P}}$ as follows. Given any split $\{{{\,\mathrm{\textit{Le}}\,}}(P_u), {{\,\mathrm{\textit{Le}}\,}}(P_v)\}$, without loss of generality, we assume that the leaf *l* occurs in the $P_u$ side of this split, i.e., $l \in {{\,\mathrm{\textit{Le}}\,}}(P_u)$, and define $f(\{{{\,\mathrm{\textit{Le}}\,}}(P_u), {{\,\mathrm{\textit{Le}}\,}}(P_v)\}) = C_{{\widehat{P}}}(v)$.

Note that $RF (P, Q)$ is equal to $2 \times (|{{\,\mathrm{\textit{Split}}\,}}(P) \setminus {{\,\mathrm{\textit{Split}}\,}}(Q)|)$. Likewise, $RF ({\widehat{P}}, {\widehat{Q}})$ is equal to $2 \times (|{{\,\mathrm{\textit{Clade}}\,}}({\widehat{P}}) \setminus {{\,\mathrm{\textit{Clade}}\,}}({\widehat{Q}})|)$. It therefore suffices to show that, given any split $\{X, Y\}$ from *P*, $\{X, Y\} \in {{\,\mathrm{\textit{Split}}\,}}(Q)$ if and only if $f(\{X,Y\}) \in {{\,\mathrm{\textit{Clade}}\,}}({\widehat{Q}})$. Suppose $\{X, Y\} \in {{\,\mathrm{\textit{Split}}\,}}(Q)$. Without loss of generality, we may assume that $l \in X$. This implies that $f(\{X,Y\}) = Y$. Since $\{X, Y\} \in {{\,\mathrm{\textit{Split}}\,}}(Q)$, there must be a node $q \in V({\widehat{Q}})$ such that $C_{{\widehat{Q}}}(q) = Y$. Thus, $f(\{X,Y\}) = C_{{\widehat{Q}}}(q)$, and so $f(\{X,Y\}) \in {{\,\mathrm{\textit{Clade}}\,}}({\widehat{Q}})$. Conversely, suppose $\{X, Y\} \not \in {{\,\mathrm{\textit{Split}}\,}}(Q)$. Again, without loss of generality, we may assume that $l \in X$ and so $f(\{X,Y\}) = Y$. There cannot be any edge $(u, v) \in E(Q)$ for which either $Q_u$ or $Q_v$ is equal to *Y*. Thus, there cannot be any node *q* in $V({\widehat{Q}})$ for which $C_{{\widehat{Q}}}(q) = Y$. Thus, $f(\{X,Y\}) \not \in {{\,\mathrm{\textit{Clade}}\,}}({\widehat{Q}})$.

$$\square$$

### **Lemma 2**

*LetSandTbe unrooted trees such that*${{\,\mathrm{\textit{Le}}\,}}(T) \subseteq {{\,\mathrm{\textit{Le}}\,}}(S)$. *Let*$T'$*be an optimal completion ofTon*${{\,\mathrm{\textit{Le}}\,}}(S)$, *such that*$T'$*minimizes*$RF (S, T')$. *Letlbe any leaf node common toTandS*. *Let*${\widehat{S}}$*be obtained by rootingSon the edge connectinglto the rest ofS*, *and*${\widehat{T}}$*be obtained by rootingTon the edge connectinglto the rest ofT*. *If*${\widehat{T}}'$*is an optimal completion of*${\widehat{T}}$*on*${{\,\mathrm{\textit{Le}}\,}}({\widehat{S}})$*then*$RF (S, T') = RF ({\widehat{S}}, {\widehat{T}}')$.

### *Proof*

Observe that *S* and $T'$ are on the same leaf set. Let $T''$ be obtained by rooting $T'$ on the edge connecting *l* to the rest of $T'$. The tree $T''$ must be a valid (not necessarily optimal) completion of the tree ${\widehat{T}}$ on ${{\,\mathrm{\textit{Le}}\,}}({\widehat{S}})$. Thus, by Observation 1, $RF (S, T') = RF ({\widehat{S}}, T'')$.

Likewise, observe that ${\widehat{S}}$ and ${\widehat{T}}'$ are on the same leaf set. Let ${\widehat{T}}''$ be the unrooted tree obtained by suppressing the root node of ${\widehat{T}}'$. The tree ${\widehat{T}}''$ must be a valid (not necessarily optimal) completion of the tree *T* on ${{\,\mathrm{\textit{Le}}\,}}(S)$. Thus, by Observation 1, $RF ({\widehat{S}}, {\widehat{T}}') = RF (S, {\widehat{T}}'')$.

We claim that $T''$ must be an optimal completion of ${\widehat{T}}$ on ${{\,\mathrm{\textit{Le}}\,}}({\widehat{S}})$. If not, then $RF ({\widehat{S}}, {\widehat{T}}') < RF ({\widehat{S}}, T'')$, implying that $RF (S, {\widehat{T}}'') < RF (S, T')$, which is a contradiction since $T'$ is an optimal completion of *T* on ${{\,\mathrm{\textit{Le}}\,}}(S)$. Thus, we must have $RF ({\widehat{S}}, {\widehat{T}}') = RF ({\widehat{S}}, T'')$, implying that $RF (S, T') = RF ({\widehat{S}}, {\widehat{T}}')$. $\square$

Based on the observation above, we solve the UOT-RF(+) problem as follows:

**Algorithm for UOT-RF(+) on input trees***S***and***T*:

1. Let *l* be any leaf from ${{\,\mathrm{\textit{Le}}\,}}(T)$. Construct ${\widehat{S}}$ by rooting *S* on the edge connecting *l* to the rest of *S*, and ${\widehat{T}}$ by rooting *T* on the edge connecting *l* to the rest of *T*.
2. Call Algorithm $\textit{OneTreeCompletion}$ with trees ${\widehat{S}}$ and ${\widehat{T}}$ as input. Let ${\widehat{T}}'$ be the tree returned.
3. Convert ${\widehat{T}}'$ into an unrooted tree by suppressing the root node and output the resulting tree.

### **Theorem 2**

*The UOT-RF(+) problem can be solved inO*(|*V*(*S*)|) *time.*

### *Proof*

Let $T^*$ denote the output of the algorithm described above, and let $T'$ denote an optimal completion of *T* on ${{\,\mathrm{\textit{Le}}\,}}(S)$. Since ${\widehat{S}}$ and ${\widehat{T}}$ are rooted at a common leaf-edge, *l*, of *S* and *T*, and since the tree ${\widehat{T}}'$ minimizes $RF ({\widehat{S}}, {\widehat{T}}')$, Lemma 2 implies that $RF (S, T') = RF ({\widehat{S}}, {\widehat{T}}')$.

Now, observe that *S* and $T^*$ have the same leaf set, and that *l* is a leaf node common to *S* and $T^*$. Furthermore, ${\widehat{S}}$ is obtained by rooting *S* on the edge connecting *l* to the rest of *S*, and ${\widehat{T}}'$ is obtained by rooting $T^*$ on the edge connecting *l* to the rest of $T^*$. Thus, by Observation 1, we must have $RF (S, T^*) = RF ({\widehat{S}}, {\widehat{T}}')$. Thus, $RF (S, T^*)$ must be equal to $RF (S, T')$, implying that $T^*$ is an optimal completion of *T* on ${{\,\mathrm{\textit{Le}}\,}}(S)$. $\square$

The previous fastest algorithm for solving the UOT-RF(+) problem [28] has quadratic time complexity. Our algorithm is able to find edges on which to graft the missing subtrees more efficiently than the algorithm from [28] because we use appropriately rooted versions of the unrooted input trees and then use simple post-order and pre-order tree traversals of the trees coupled with efficient least common ancestor computations.

## The R-RF(+) problem

**Fig. 3:**
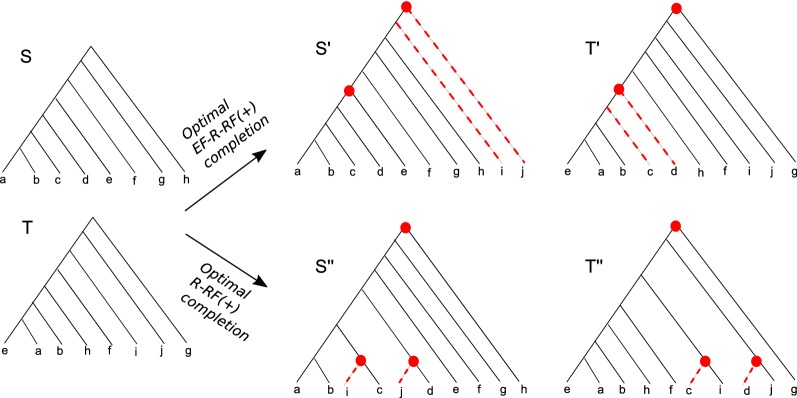
Extraneous clades and R-RF(+) and EF-R-RF(+) completions. This figure shows two trees *S* and *T* with partial leaf set overlap whose optimal completions under the R-RF(+) problem result in extraneous clades. The tree *S* contains two leaves *c* and *d* that are absent from *T*, and the tree *T* contains two leaves *i* and *j* absent from *S*. The lower-right part of the figure shows optimal completions of *S* and *T*, labeled $S''$ and $T''$, respectively, that minimize the RF distance over all possible completions. The nodes marked in red denote (non-leaf) clades common to both $S''$ and $T''$. Observe that of the three nodes that $S''$ and $T''$ have in common, the lower two, i.e., $\{c, i\}$ and $\{d, j\}$, are extraneous clades that have no support in either *S* or *T* and do not contain any of the leaves shared by both *S* and *T*. Optimal completions under EF-R-RF(+) disallow such extraneous clades. The upper-right part of the figure shows optimal completions of *S* and *T* that minimize the RF distance over all completions without any extraneous clades. The completions $S'$ and $T'$ only contain clades that have at least one leaf shared by both trees

Observe how an optimal completion of *T* in the ROT-RF(+) problem maximizes the number of clades that have a match in *S*. This ensures a meaningful completion of *T*. However, in the R-RF(+) problem, where both trees may have missing leaves, it is possible that optimal completions of the two trees contain “extraneous” clades that contain leaves from both *S* and *T* but do not contain any leaves common to *S* and *T*. Extraneous clades are created by pairing a subtree containing only missing leaves from one tree with a subtree containing only missing leaves from the other tree. Such clades can help to lower the RF distance between the two completed trees, but are completely unsupported by the topologies of *S* and *T*. This phenomenon is illustrated through an example in Fig. 3. We therefore define a variant of the R-RF(+) problem that only allows completions that do not result in extraneous clades. Crucially, this restriction to only non-extraneous clades also makes the underlying completion problem easier to solve. Note that extraneous clades could indeed be “correct”

in some cases, so restricting to non-extraneous clades could sometimes prevent us from considering certain correct clades when computing completions.

### **Definition 5**

(Extraneous clade) Suppose *S* and *T* are rooted trees. Given completions $S'$ and $T'$ of *S* and *T*, respectively, on ${{\,\mathrm{\textit{Le}}\,}}(S) \cup {{\,\mathrm{\textit{Le}}\,}}(T)$, we define a clade of $S'$ or $T'$ to be an *extraneous clade* if it contains leaves from both *S* and *T* but no leaves that are common to *S* and *T*.

### **Problem 5**

(Extraneous-Clade-Free R-RF(+) (EF-R-RF(+))) *Given two rooted treesSandT*, *compute a completion*$S'$*ofSon*${{\,\mathrm{\textit{Le}}\,}}(S) \cup {{\,\mathrm{\textit{Le}}\,}}(T)$*and a completion*$T'$*ofTon*${{\,\mathrm{\textit{Le}}\,}}(S) \cup {{\,\mathrm{\textit{Le}}\,}}(T)$*such that*$S'$*and*$T'$*do not contain any extraneous clades and*$RF (S', T')$*is minimized.*

An example of an optimal EF-R-RF(+) completion appears in Fig. 3. Next, we show how to solve the EF-R-RF(+) problem in linear time.

### A linear-time algorithm for EF-R-RF(+)

For the EF-R-RF(+) problem, ${{\,\mathrm{\textit{Le}}\,}}(S)$ and ${{\,\mathrm{\textit{Le}}\,}}(T)$ are both proper subsets of ${{\,\mathrm{\textit{Le}}\,}}(S) \cup {{\,\mathrm{\textit{Le}}\,}}(T)$, i.e., both *S* and *T* must be completed on the leaf set ${{\,\mathrm{\textit{Le}}\,}}(S) \cup {{\,\mathrm{\textit{Le}}\,}}(T)$. Our algorithm for this problem builds upon the algorithm for the ROT-RF(+) problem. Specifically, we first complete *T* on ${{\,\mathrm{\textit{Le}}\,}}(S) \cup {{\,\mathrm{\textit{Le}}\,}}(T)$ with respect to *S*, then complete *S* on ${{\,\mathrm{\textit{Le}}\,}}(S) \cup {{\,\mathrm{\textit{Le}}\,}}(T)$ with respect to the previous completion of *T*. Formally, the algorithm is as follows: 
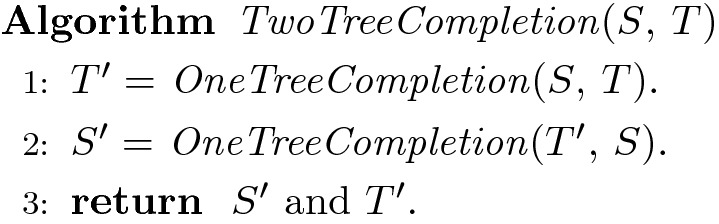


In the following, we will show that when Algorithm *TwoTreeCompletion* terminates, the trees $S'$ and $T'$ returned by the algorithm must be such that they do not contain any extraneous clades, and that $RF (S', T')$ is the smallest possible for any completion of *S* and *T* that does not have extraneous clades. We will assume, without any loss of generality, that *S* and *T* have at least one leaf in common; if there are no leaves in common between *S* and *T* then the EF-R-RF(+) problem has no solution since any completion of *S* and *T* would necessarily contain extraneous clades.

For brevity, in the remainder of this section, we will implicitly assume that all completions of *S* and *T* are on the leaf set ${{\,\mathrm{\textit{Le}}\,}}(S) \cup {{\,\mathrm{\textit{Le}}\,}}(T)$. Next, we define the notions of *original nodes*, *grafted nodes*, and *grafted subtrees* in tree completions.

#### **Definition 6**

(Original nodes) Let $S'$ and $T'$ denote any completions of *S* and *T*. Observe that completing a tree creates new internal nodes in the tree but preserves all original internal nodes (though not necessarily the clades rooted at those nodes). Thus, we have $I(S) \subset I(S')$ and $I(T) \subset I(T')$. The set of nodes in $I(S')$ that are also present in *I*(*S*) are called the *original nodes* of $S'$, denoted ${\mathcal {O}}(S')$. Analogously, the set of nodes in $I(T')$ that are also present in *I*(*T*) are called the *original nodes* of $T'$, denoted ${\mathcal {O}}(T')$.

#### **Definition 7**

(Grafted nodes) Let $S'$ and $T'$ denote any completions of *S* and *T*. Observe that any node $u \in I(S') \setminus {\mathcal {O}}(S')$ is either a node that was already present in a subtree from *T* (consisting of leaves missing from *S*) as that subtree was grafted into *S*, or a new node that was created as a subtree from *T* (consisting of leaves missing from *S*) was grafted into *S*. We refer to the new nodes created by the grafting of a subtree from *T* into $S'$ as the *grafted nodes* of $S'$, denoted ${\mathcal {G}}(S')$. Analogously, the set of nodes in $I(T') \setminus {\mathcal {O}}(T')$ that were newly created through the process of grafting a subtree from *S* into *T* are called the *grafted nodes* of $T'$, denoted ${\mathcal {G}}(T')$.

#### **Definition 8**

(Grafted subtrees) If $S'$ denotes any completion of *S* and $u \in {\mathcal {G}}(S')$, then *u* is created by the grafting of a subtree of *T* (consisting of leaves missing from *S*) at that node *u* in $S'$. We denote the grafted subtree of *T* at *u* by $\textit{graft}(u)$. Similarly, if $T'$ denotes any completion of *T* and $v \in {\mathcal {G}}(T')$, then *v* is created by the grafting of a subtree of *S* at that node *v* in $T'$. We denote the grafted subtree of *S* at *v* by $\textit{graft}(v)$.

### Node colorings

For convenience, we will color the nodes of *S* and *T* according to the coloring scheme used in Algorithm *OneTreeCompletion*. Thus, each node of *S* and *T* is colored either red, or green, or blue. We will assume that these colored nodes maintain their original colors in the completed trees $S'$ and $T'$, and thus both $S'$ and $T'$ contain nodes that are red, green, and blue, as well as nodes that are uncolored.

We now show that the completed trees $S'$ and $T'$ returned by Algorithm *TwoTreeCompletion* must be free of extraneous clades.

#### **Lemma 3**

*The trees* $S'$*and* $T'$*returned by AlgorithmTwoTreeCompletiondo not have any extraneous clades.*

#### *Proof*

Let us first consider the tree $T'$. Any non-original node in $T'$ is either a node from a maximal red subtree of *S* or is a grafted node created by grafting a maximal red subtree of *S* into $T'$ using the $\textit{Tree-Add}$ operation. Based on Algorithm *OneTreeCompletion*, each grafted node created through the *Tree-Add* operation has at least one green descendant, and so it cannot be extraneous. Moreover, any node inside a maximal red subtree of *S* only has descendants from *S*, not from *T*. Thus, since *T* did not contain any extraneous clades to begin with, neither can $T'$. An analogous argument applies to $S'$. $\square$

The next lemma identifies an important property of optimal completions.

#### **Lemma 4**

*Let*$S^*$*and*$T^*$*be any optimal completions ofSandT*, *respectively, under the EF-R-RF(+) problem. Then, for any*$u \in {\mathcal {G}}(S^*)$, $\textit{graft}(u)$*must be a maximal red subtree ofTand, for any*$v \in {\mathcal {G}}(T^*)$, $\textit{graft}(v)$*must be a maximal red subtree ofS*.

#### *Proof*

Observe that any maximal red subtree of *T* must appear as is in the tree $T^*$, since grafting a red leaf or subtree from *S* into any of the red subtrees of *T* would result in an extraneous clade. We will show that if there exists a node $u \in {\mathcal {G}}(S^*)$ for which $\textit{graft}(u)$ is not a maximal red subtree of *T*, it is possible to modify the tree $S^*$ so that the modified tree has more matched clades than $S^*$, a contradiction. An analogous argument applies to $T^*$. Suppose there exists such a node *u*. Then, there must exist a red internal node *r* of *T* such that the two subtrees, denoted $R'$ and $R''$, rooted at the two children of *r* appear as is in the tree $S^*$ but not as siblings of each other (i.e., their roots do not have the same parent in $S^*$). Let $r'$ and $r''$ denote the root nodes of $R'$ and $R''$, respectively, and $s'$ and $s''$ denote the parents of $r'$ and $r''$ in $S^*$. Thus, $R' = \textit{graft}(s')$ and $R'' = \textit{graft}(s'')$. Now, observe that all clades of $S^*$ rooted either at a node on the path from ${{\,\mathrm{\textit{lca}}\,}}_{S^*}(s', s'')$ to $s'$ or on the path from ${{\,\mathrm{\textit{lca}}\,}}_{S^*}(s', s'')$ to $s''$, except for the node ${{\,\mathrm{\textit{lca}}\,}}_{S^*}(s', s'')$ itself, must be mismatched clades (since all maximal red subtrees of *T* appear as is in the tree $T^*$). Also, note that if $S^*$ is modified by pruning out the subtree $R'$ and regrafting it on the edge $(s'', r'')$ then the only matched clades that can become mismatched are the ones whose roots lie on the path from ${{\,\mathrm{\textit{lca}}\,}}_{S^*}(s', s'')$ to $s'$ or from ${{\,\mathrm{\textit{lca}}\,}}_{S^*}(s', s'')$ to $s''$, except for node ${{\,\mathrm{\textit{lca}}\,}}_{S^*}(s', s'')$. Thus, modifying the tree $S^*$ in this fashion does not result in any additional mismatched clades, but results in a new matched clade rooted at the node where $R'$ is regrafted. Thus, the modified tree has a larger number of matched clades than $S^*$, which is a contradiction. $\square$

We also have the following simple observation about optimal completions.

#### **Observation 2**

*Let*$S^*$*and*$T^*$*be optimal completions ofSandT*, *respectively, that satisfy the property described in Lemma* 4. *Then any*$u \in {\mathcal {G}}(S^*)$*and any*$v \in {\mathcal {G}}(T^*)$ must *have at least one green leaf as a descendant.*

#### *Proof*

This follows immediately from the fact that, under EF-R-RF(+), each clade must contain at least one green leaf (otherwise it would be an extraneous clade). $\square$

Finally, the following lemma proves the correctness of Algorithm *TwoTreeCompletion*.

#### **Lemma 5**

*Let*$S'$*and*$T'$*denote the completions ofSandT*, *respectively, returned by Algorithm* *TwoTreeCompletion*. *Let*$S^*$*and*$T^*$*denote optimal completions ofSandT*, *respectively, under the EF-R-RF(+) problem. Then,*$RF (S', T') = RF (S^*, T^*)$.

#### *Proof*

Based on Lemma 4, we know that $S^*$ and $T^*$ are such that, for any $u \in {\mathcal {G}}(S^*)$, $\textit{graft}(u)$ is a maximal red subtree of *T*, and for any $v \in {\mathcal {G}}(T^*)$, $\textit{graft}(v)$ is a maximal red subtree of *S*. Furthermore, observe that, given the tree $T^*$, we can compute an optimal completion for *S* with respect to $T^*$ by using Algorithm *OneTreeCompletion*. Thus, without any loss of generality, we will assume that $S^*$ has the topology that would be computed by Algorithm *OneTreeCompletion* on input $(T^*, S)$.

To prove this lemma, it suffices to show that the number of matched clades in $T'$ (with respect to $S'$) is no less than the number of matched clades in $T^*$ (with respect to $S^*$). We first define a one-to-one correspondence between the internal nodes of $T'$ and the internal nodes of $T^*$. Consider any node $t \in I(T')$. There are three possibilities: (i) $t \in {\mathcal {O}}(T')$, (ii) $t \in {\mathcal {G}}(T')$, and (iii) *t* is a node from a maximal red subtree of *S*. For case (i), observe that ${\mathcal {O}}(T') = {\mathcal {O}}(T^*)$, and so if $t \in {\mathcal {O}}(T')$ then a counterpart of *t* also exists in $T^*$. For case (ii) observe that each $t \in {\mathcal {G}}(T')$ is created by grafting $\textit{graft}(t)$ into $T'$. We will associate *t* with that unique node of $T^*$ that is created by grafting the same maximal red subtree of *S*, $\textit{graft}(t)$, into $T^*$. For case (iii), since the same maximal red subtree of *S* also appears in $T^*$, the node associated with *t* is the same node from the same maximal red subtree of *S* in $T^*$. We denote the node of $I(T^*)$ corresponding to node $t \in I(T')$ by *f*(*t*). It is not difficult to see that $f :I(T') \rightarrow I(T^*)$ is one-to-one and onto.

We now traverse the nodes of $T'$ in post order and identify the first node $t \in I(T')$ for which $C_{T'}(t)$ is a mismatch in $S'$ but $C_{T^*}(f(t))$ is a match in $S^*$. If no such node exists then the number of matched clades in $T^*$ could not be more than the number of matched clades in $T'$, completing our proof. Thus, suppose such a node *t* exists. We again have three possible cases depending on whether (i) $t \in {\mathcal {O}}(T')$, (ii) $t \in {\mathcal {G}}(T')$, or (iii) *t* is a node from a maximal red subtree of *S*. We consider each of these cases separately.

*Case (i):*$t \in {\mathcal {O}}(T')$. In this case, $C_{T'}(t)$ must be a proper subset of $C_{T^*}(f(t))$. This is because if $C_{T'}(t) = C_{T^*}(f(t))$ then both clades would either be matches or both would be mismatches, while if $T'(t)$ contains a grafted subtree not present in $T^*(f(t))$ then $C_{T^*}(f(t))$ could not possibly be a matched clade. Thus, there must be at least one maximal red subtree of *S* that is grafted on an edge in $T^*(f(t))$ but not on an edge in $T'(t)$. We let *X* denote the set of such maximal red subtrees, and let $G^*$ denote the set of grafted nodes corresponding to these maximal red subtrees from *X* in the tree $T^*$.

Let *a* be any node on the path from *f*(*t*) to any $g \in G^*$ in $T^*$, except for the node *f*(*t*) itself. Since $T'$ is computed by executing Algorithm *OneTreeCompletion* on input (*S*, *T*), and no subtree from *X* is grafted inside the subtree $T'(t)$, $T^*(a)$ cannot be a matched clade. We can therefore modify $T^*$ by cutting out all subtrees of *X* from $T^*(f(t))$ and grafting them onto the parent edge of *f*(*t*) (in any arbitrary order if $|X| > 1$). Let $T^*_M$ denote this modified version of $T^*$, and let $g^*$ denote the newly created grafted node that is closest to ${{\,\mathrm{\textit{rt}}\,}}(T^*_M)$ along the path from ${{\,\mathrm{\textit{rt}}\,}}(T^*_M)$ to *f*(*t*) in $T^*_M$. Observe that $C_{T^*_M}(g^*) = C_{T^*}(f(t))$, and so $C_{T^*_M}(g^*)$ must be a matched clade in $T^*_M$, while $C_{T^*_M}(f(t))$ is no longer a matched clade. Thus, overall, the number of matched clades in $T^*_M$ is the same as the number of matched clades in $T^*$. If we now assign $T^*$ to be $T^*_M$ then node *t* is no longer such that $C_{T'}(t)$ is a mismatch in $S'$ but $C_{T^*}(f(t))$ is a match in $S^*$. Moreover, observe that any grafted node corresponding to a maximal red subtree from *X* in the tree $T'$ must lie along the path from ${{\,\mathrm{\textit{rt}}\,}}(T')$ to *t* (otherwise $C_{T^*}(f(t))$ could not be a matched clade). Thus, the nodes of $I(T')$ that have already been considered so far in the post-order traversal remain unaffected by the change in the topology of $T^*$.

*Case (ii):*$t \in {\mathcal {G}}(T')$. The argument in this case is similar to that from case (i). As before, $C_{T'}(t)$ must be a proper subset of $C_{T^*}(f(t))$. This is because if $C_{T'}(t) = C_{T^*}(f(t))$ then both clades would either be matches or both would be mismatches, while if $T'(t)$ contains a grafted subtree not present in $T^*(f(t))$ then $C_{T^*}(f(t))$ could not possibly be a matched clade. There are therefore two possibilities: $T^*(f(t))$ either includes an original node $r \in {\mathcal {O}}(T^*)$ for which the corresponding original node in $T'$ is an ancestor of *t*, or $T^*(f(t))$ does not include such an original node.

For the first possibility, $T^*(f(t))$ includes an original node $r \in {\mathcal {O}}(T^*)$ for which the corresponding original node in $T'$ is an ancestor of *t*. Without loss of generality, let *r* denote that original node from $T^*(f(t))$ that is closest to *f*(*t*). Let *a* be any node along the path from *f*(*t*) to *r*, except for *f*(*t*) itself. Observe that *a* must be a grafted node and that $C_{T^*}(a)$ cannot be a match since it does not include the subtree $\textit{graft}(t)$. We can therefore modify $T^*$ by cutting out all grafted subtrees along the path from *f*(*t*) to *r* (including $\textit{graft}(t)$) and grafting them in the same order onto any chosen child edge of *r*. Let $T^*_M$ denote this modified version of $T^*$. Note that $C_{T^*_M}(r) = C_{T^*}(f(t))$, and so $C_{T^*_M}(r)$ must be a matched clade in $T^*_M$, while $C_{T^*_M}(f(t))$ is no longer a matched clade. Note, also, that the other newly formed clades in $C_{T^*_M}(f(t))$ must all be mismatches since $T^*_M$ cannot have more matched clades than the optimal completion $T^*$. Thus, overall, the number of matched clades in $T^*_M$ is the same as the number of matched clades in $T^*$. If we now assign $T^*$ to be $T^*_M$ then node *t* is no longer such that $C_{T'}(t)$ is a mismatch in $S'$ but $C_{T^*}(f(t))$ is a match in $S^*$, while the nodes of $I(T')$ that have already been considered so far in the post-order traversal also remain unaffected by the change in the topology of $T^*$ (since the original node corresponding to *r* in $T'$ is an ancestor of *t*).

For the second possibility, $T^*(f(t))$ must include a grafted subtree (maximal red subtree of *S*) that is not present in $T'(t)$. Let *g* denote the grafted node of $T^*(f(t))$ at which any such subtree is grafted, and let *a* be any node on the path from *f*(*t*) to *g* in $T^*$, except for the node *f*(*t*) itself. Note that, since $T'$ is computed by executing Algorithm *OneTreeCompletion* on input (*S*, *T*), and this maximal red subtree is not grafted inside the subtree $T'(t)$, $T^*(a)$ cannot be a matched clade. We can therefore modify $T^*$ by cutting out this grafted subtree at *g* and grafting it at the parent edge of *f*(*t*) (creating such an edge *f*(*t*) happens to be the root of $T^*$). Let the new node thus created be called $g^*$ and let $T^*_M$ denote this modified version of $T^*$. Note that $C_{T^*_M}(g^*) = C_{T^*}(f(t))$, and so $C_{T^*_M}(g^*)$ must be a matched clade in $T^*_M$, while $C_{T^*_M}(f(t))$ is no longer a matched clade. Note, also, that no other matched clades of $T^*$ are affected by this transformation. Thus, overall, the number of matched clades in $T^*_M$ is the same as the number of matched clades in $T^*$. Furthermore, observe that $\textit{graft}(g)$ must be a subtree that appears grafted somewhere along the path from *t* to ${{\,\mathrm{\textit{rt}}\,}}(T')$ in tree $T'$, since otherwise, for $C_{T^*}(f(t))$ to be a match, $T^*(f(t))$ would have to contain an original node $r \in {\mathcal {O}}(T^*)$ for which the corresponding original node in $T'$ is an ancestor of *t*, a contradiction of the premise of this second possibility. If we now assign $T^*$ to be $T^*_M$ then node *t* is no longer such that $C_{T'}(t)$ is a mismatch in $S'$ but $C_{T^*}(f(t))$ is a match in $S^*$, while the nodes of $I(T')$ that have already been considered so far in the post-order traversal also remain unaffected by the change in the topology of $T^*$.

*Case (iii): t is a node from a maximal red subtree of S*. Observe that if *t* is a node from a maximal red subtree of *S* then $C_{T'}(t)$ will always be a match in $S'$. This is because the maximal red subtree of *S* that contains *t* appears as is in $S'$. Thus, *t* could not have been a node from a maximal red subtree of *S*, and so this case never arises.

A simple inductive argument based on the post-order traversal of $T'$ now completes this proof. $\square$

The next theorem now follows immediately based on Algorithm *TwoTreeCompletion*, Theorem 1, and Lemma 5.

#### **Theorem 3**

*Algorithm* *TwoTreeCompletionsolves the EF-R-RF(+) problem in*$O(|V(S)| + |V(T)|)$*time.*

## The EF-U-RF(+) problem

Observe that if *S* and *T* are unrooted and $|{{\,\mathrm{\textit{Le}}\,}}(S) \cap {{\,\mathrm{\textit{Le}}\,}}(T)| < 2$ then there do not exist any completions $S'$ and $T'$ of *S* and *T*, respectively, that do not contain an extraneous clade when $S'$ and $T'$ are rooted using a leaf node from ${{\,\mathrm{\textit{Le}}\,}}(S) \cap {{\,\mathrm{\textit{Le}}\,}}(T)$. Thus, we will assume that $|{{\,\mathrm{\textit{Le}}\,}}(S) \cap {{\,\mathrm{\textit{Le}}\,}}(T)| \ge 2$. We first define the concept of an extraneous split and then define the EF-U-RF(+) problem.

### **Definition 9**

(Extraneous split) Suppose *S* and *T* are unrooted trees. Let *l* be any leaf from ${{\,\mathrm{\textit{Le}}\,}}(S) \cap {{\,\mathrm{\textit{Le}}\,}}(T)$, and $S'$ and $T'$ be completions of *S* and *T*, respectively, on ${{\,\mathrm{\textit{Le}}\,}}(S) \cup {{\,\mathrm{\textit{Le}}\,}}(T)$. Let ${\widehat{S}}'$ be obtained by rooting $S'$ on the edge connecting *l* to the rest of $S'$, and ${\widehat{T}}'$ be obtained by rooting $T'$ on the edge connecting *l* to the rest of $T'$. We define a split of $S'$ or $T'$ to be an *extraneous split* if the corresponding clade in ${\widehat{S}}'$ or ${\widehat{T}}'$ is an extraneous clade.

### **Problem 6**

(Extraneous-Split-Free U-RF(+) (EF-U-RF(+))) *Given two unrooted treesSandTsuch that*$|{{\,\mathrm{\textit{Le}}\,}}(S) \cap {{\,\mathrm{\textit{Le}}\,}}(T)| \ge 2$, *compute a completion*$S'$*ofSon*${{\,\mathrm{\textit{Le}}\,}}(S) \cup {{\,\mathrm{\textit{Le}}\,}}(T)$ and a completion $T'$ of *T* on ${{\,\mathrm{\textit{Le}}\,}}(S) \cup {{\,\mathrm{\textit{Le}}\,}}(T)$*such that*$S'$*and*$T'$*do not contain any extraneous splits and*$RF (S', T')$*is minimized.*

As in Section 4, we will show how to solve EF-U-RF(+) by solving EF-R-RF(+). In particular, we solve the EF-U-RF(+) problem using the following algorithm.

**Algorithm for EF-U-RF(+) on input trees ***S*** and ***T***:**

1. Let *l* be any leaf from ${{\,\mathrm{\textit{Le}}\,}}(S) \cap {{\,\mathrm{\textit{Le}}\,}}(T)$. Construct ${\widehat{S}}$ by rooting *S* on the edge connecting *l* to the rest of *S*, and ${\widehat{T}}$ by rooting *T* on the edge connecting *l* to the rest of *T*.
2. Call Algorithm $\textit{TwoTreeCompletion}$ with trees ${\widehat{S}}$ and ${\widehat{T}}$ as input. Let ${\widehat{S}}'$ and ${\widehat{T}}'$ be the trees returned.
3. Convert ${\widehat{S}}'$ and ${\widehat{T}}'$ into unrooted trees by suppressing the root node and output the resulting trees.

We will show that the completed unrooted trees, $S'$ and $T'$, returned by the above algorithm must be extraneous-split-free and minimize $RF (S', T')$.

### **Lemma 6**

*The trees* $S'$*and* $T'$*returned by the above algorithm do not have any extraneous splits.*

### *Proof*

Since the trees ${\widehat{S}}'$ and ${\widehat{T}}'$ computed in the above algorithm do not have any extraneous clades, each clade in ${{\,\mathrm{\textit{Clade}}\,}}({\widehat{S}}')$ and ${{\,\mathrm{\textit{Clade}}\,}}({\widehat{T}}')$ must have at least one leaf from ${{\,\mathrm{\textit{Le}}\,}}(S) \cap {{\,\mathrm{\textit{Le}}\,}}(T)$. Now, consider any leaf $l'\in {{\,\mathrm{\textit{Le}}\,}}(S) \cap {{\,\mathrm{\textit{Le}}\,}}(T)$, and let ${\widehat{S}}''$ be obtained by rooting $S'$ on the edge connecting $l'$ to the rest of $S'$, and ${\widehat{T}}''$ be obtained by rooting $T'$ on the edge connecting $l'$ to the rest of $T'$. Observe that any clade in ${{\,\mathrm{\textit{Clade}}\,}}({\widehat{S}}'')$ must either be a clade from ${{\,\mathrm{\textit{Clade}}\,}}({\widehat{S}}')$ or must contain the leaf *l*. Likewise, any clade in ${{\,\mathrm{\textit{Clade}}\,}}({\widehat{T}}'')$ must either be a clade from ${{\,\mathrm{\textit{Clade}}\,}}({\widehat{T}}')$ or must contain the leaf *l*. Thus, neither ${\widehat{S}}''$ nor ${\widehat{T}}''$ contain any extraneous clades, and so, by the definition of an extraneous split, the trees $S'$ and $T'$ must be free of any extraneous splits. $\square$

### **Lemma 7**

*LetSandTbe unrooted trees with partially overlapping leaf sets and*$|{{\,\mathrm{\textit{Le}}\,}}(S) \cap {{\,\mathrm{\textit{Le}}\,}}(T)| \ge 2$. *Let*$S'$*be an optimal completion ofSand*$T'$*be an optimal completion ofT*, *on*${{\,\mathrm{\textit{Le}}\,}}(T) \cup {{\,\mathrm{\textit{Le}}\,}}(S)$, *such that*$S'$*and*$T'$*do not contain any extraneous splits and minimize*$RF (S', T')$. *Letlbe any leaf node common toSandT*. *Let*${\widehat{S}}$*be obtained by rootingSon the edge connectinglto the rest ofS*, *and*${\widehat{T}}$*be obtained by rootingTon the edge connectinglto the rest ofT*. *If*${\widehat{S}}'$*and*${\widehat{T}}'$*are optimal completions of*${\widehat{S}}$*and*${\widehat{T}}$, *respectively, under the EF-R-RF(+) problem, then*$RF (S', T') = RF ({\widehat{S}}', {\widehat{T}}')$.

### *Proof*

Observe that $S'$ and $T'$ are on the same leaf set. Let $T''$ be obtained by rooting $T'$ on the edge connecting *l* to the rest of $T'$, and $S''$ be obtained by rooting $S'$ on the edge connecting *l* to the rest of $S'$. The trees $T''$ and $S''$ must be valid (but not necessarily optimal) completions of the trees ${\widehat{T}}$ and ${\widehat{S}}$ under the EF-R-RF(+) problem. Thus, by Observation 1, $RF (S', T') = RF (S'', T'')$.

Likewise, observe that ${\widehat{S}}'$ and ${\widehat{T}}'$ are on the same leaf set. Let ${\widehat{S}}''$ and ${\widehat{T}}''$ be the unrooted trees obtained by suppressing the root nodes of ${\widehat{S}}'$ and ${\widehat{T}}'$, respectively. As shown in Lemma 6, the trees ${\widehat{S}}''$ and ${\widehat{T}}''$ must be valid (not necessarily optimal) completions of *S* and *T* under the EF-U-RF(+) problem. Thus, by Observation 1, $RF ({\widehat{S}}', {\widehat{T}}') = RF ({\widehat{S}}'', {\widehat{T}}'')$.

We claim that $S''$ and $T''$ must be optimal completions of ${\widehat{S}}$ and ${\widehat{T}}$, respectively, on ${{\,\mathrm{\textit{Le}}\,}}(T) \cup {{\,\mathrm{\textit{Le}}\,}}(S)$. If not, then $RF ({\widehat{S}}', {\widehat{T}}') < RF (S'', T'')$, implying that $RF ({\widehat{S}}'', {\widehat{T}}'') < RF (S', T')$, which is a contradiction since $S'$ and $T'$ are optimal completions of *S* and *T* under the EF-U-RF(+) problem. Thus, we must have $RF ({\widehat{S}}', {\widehat{T}}') = RF (S'', T'')$, implying that $RF (S', T') = RF ({\widehat{S}}', {\widehat{T}}')$. $\square$

Lemma 7 proves that the algorithm described above correctly solves the EF-U-RF(+) problem. Furthermore, note that the time complexity of the algorithm above is dominated by the time complexity of Algorithm *TwoTreeCompletion*, which is $O(|V(S)| + |V(T)|)$. Thus, we immediately have the following theorem.

### **Theorem 4**

*The EF-U-RF(+) problem can be solved in* $O(|V(S)| + |V(T)|)$*time.*

## Experimental evaluation

We implemented our algorithm for the ROT-RF(+) problem and applied it to three large biological supertree data sets with the goal of assessing the impact of using RF(+) distance instead of the traditional RF(−) distance in practice. Specifically, we computed a supertree (using a standard supertree method; RFS [13] in this case) for each of the supertree data sets, and computed the RF(+) and RF(−) distances between the supertree and the input trees for each data set. Let the RF(+) distance between a supertree *S* and an input tree *I* be denoted by $RF ^{+}(S, I)$, and the RF(−) distance those two trees by $RF ^{-}(S, I)$. For each data set, we ordered the input trees according to their RF(+) and RF(−) distances to the supertree and measured how often the relative ranking between any pair of input trees differs between the two rankings. More precisely, given a supertree *S* and its set of input trees ${\mathcal {I}}$, we computed $RF ^{-}(S, I)$ and $RF ^+(S, I)$ for each $I \in {\mathcal {I}}$, and counted the number of *Type-1*, *Type-2*, and *Type-3* pairs $\{I', I''\}$, where $I', I'' \in {\mathcal {I}}$, as follows:

Type-1 pairs: Pair $\{I', I''\}$ is Type-1 if either $RF ^-(S, I') < RF ^-(S, I'')$ but $RF ^+(S, I') > RF ^+(S, I'')$, or $RF ^-(S, I') > RF ^-(S, I'')$ but $RF ^+(S, I') < RF ^+(S, I'')$. These are pairs for which the RF(+) and RF(−) distances impose completely opposite orderings relative to the supertree.
Type-2 pairs: Pair $\{I', I''\}$ is Type-2 if $RF ^-(S, I') = RF ^-(S, I'')$ but $RF ^+(S, I') \ne RF ^+(S, I'')$. For these pairs, RF(−) distances are identical but RF(+) distances are not.
Type-3 pairs: Pair $\{I', I''\}$ is Type-3 if $RF ^-(S, I') \ne RF ^-(S, I'')$ but $RF ^+(S, I') = RF ^+(S, I'')$. For these pairs, RF(+) distances are identical but RF(−) distances are not.

The three data sets, marsupials [32], placental mammals [33], and legumes [34], contain 272, 116, and 571 species, and 158, 726, and 22 input trees, respectively. We observed that for the 158 input trees of the marsupial data set, there were 521 Type-1 pairs, 619 Type-2 pairs, and 376 Type-3 pairs. For the 726 input trees of the placental mammals data set, there were 5816 Type-1 pairs, 14, 344 Type-2 pairs, and 6, 238 Type-3 pairs. Likewise, for the 22 input trees in the legumes data set, we observed 8 Type-1 pairs, 3 Type-2 pairs, and no Type-3 pairs. These results, summarized in Table 1, show that there can be substantial difference between RF(−) and RF(+) distances.

**Table 1:** Summary of results on the three datasets

	158-tree dataset	726-tree dataset	22-tree dataset
Number of Type-1 pairs	521	5816	8
Number of Type-2 pairs	619	14,344	3
Number of Type-3 pairs	376	6238	0
Total number of pairs	12,403	263,175	231
Percentage of Type-1/2/3 pairs	12.22%	10.03%	4.76%

An open-source implementation of our algorithms for ROT-RF(+) and EF-R-RF(+) is freely available from: https://compbio.engr.uconn.edu/software/rf_plus/.

## Conclusion

In this work, we provide the first optimal, linear-time algorithms for two fundamental computational problems that arise when comparing phylogenetic trees with non-identical leaf sets. For the first problem, which arises when computing the RF(+) distance between two trees where the leaf set of one tree is a proper subset of the other, we improved upon the time complexity of the previous fastest algorithm by a factor of *n*, where *n* is the number of leaves in the larger of the two trees. For the second problem, which arises when computing the RF(+) distance between two trees that have only partially overlapping leaf sets, and for which there are no existing algorithms, we defined a useful restriction of the problem and provided an optimal linear-time algorithm for it. These algorithms make it as computationally efficient to compute RF(+) distances as RF(−) distances. The algorithms work for both rooted and unrooted trees, and can be directly applied wherever phylogenetic distances must be computed between trees with non-identical leaf sets. Furthermore, our experiments with three large biological supertree data sets suggest that using the RF(+) distance can result in very different relative estimates of phylogenetic distances compared to using the RF(−) distance.

The algorithms presented here have several important, well-established applications, including construction of majority-rule(+) supertrees and supertree construction in general, phylogenetic database search, and clustering of phylogenetic trees, and these applications should be studied and developed further. A more detailed experimental study is needed to properly assess the impact of using RF(+) distances and to systematically study the effect of factors such as fraction of leaf set overlap and degree of discordance between trees. This work also motivates several theoretical questions for future investigation. For instance, our algorithms for the EF-R-RF(+) and EF-U-RF(+) problems cannot be easily extended to solve the R-RF(+) and U-RF(+) problems. In particular, if optimal completions are allowed to contain extraneous clades then inferring the number and composition of these extraneous clades (to attain overall optimality) appears to be computationally challenging. It would be interesting to determine if linear or near-linear time algorithms exist for R-RF(+) and U-RF(+).

## Data Availability

The algorithms described in this paper have been implemented in the open-source software package RF+ available freely from https://compbio.engr.uconn.edu/software/rf_plus/. The data sets used in this paper are described in [32–34].
